# Predicting hotspots for disease-causing single nucleotide variants using sequences-based coevolution, network analysis, and machine learning

**DOI:** 10.1371/journal.pone.0302504

**Published:** 2024-05-14

**Authors:** Wenjun Zheng

**Affiliations:** Department of Physics, State University of New York at Buffalo, Buffalo, NY, United States of America; Kingston University, UNITED KINGDOM

## Abstract

To enable personalized medicine, it is important yet highly challenging to accurately predict disease-causing mutations in target proteins at high throughput. Previous computational methods have been developed using evolutionary information in combination with various biochemical and structural features of protein residues to discriminate neutral vs. deleterious mutations. However, the power of these methods is often limited because they either assume known protein structures or treat residues independently without fully considering their interactions. To address the above limitations, we build upon recent progress in machine learning, network analysis, and protein language models, and develop a sequences-based variant site prediction workflow based on the protein residue contact networks: 1. We employ and integrate various methods of building protein residue networks using state-of-the-art coevolution analysis tools (RaptorX, DeepMetaPSICOV, and SPOT-Contact) powered by deep learning. 2. We use machine learning algorithms (Random Forest, Gradient Boosting, and Extreme Gradient Boosting) to optimally combine 20 network centrality scores to jointly predict key residues as hot spots for disease mutations. 3. Using a dataset of 107 proteins rich in disease mutations, we rigorously evaluate the network scores individually and collectively (via machine learning). This work supports a promising strategy of combining an ensemble of network scores based on different coevolution analysis methods (and optionally predictive scores from other methods) via machine learning to predict hotspot sites of disease mutations, which will inform downstream applications of disease diagnosis and targeted drug design.

## Introduction

The holy grail of structural biology is to solve high-resolution biomolecular structures at the genomic scale to inform mechanistic studies of their functions. Thanks to recent revolutions in computational structural biology (accurate protein structure prediction by AlphaFold [1] and RoseTTAFold [2]), it is now feasible to predict native structures for many proteins given their sequences (with some caveats, see [3]), thus practically solving the protein folding problem [4]. However, it remains challenging to predict dynamic structural ensembles [5] and mutation-induced effects [6] to meet the demand of mechanistic studies of protein functions and dysfunctions. While the public databases of protein sequences and variations increase rapidly owning to genomic/metagenomic sequencing efforts (the MetaClust database contains about 1.6 billion protein sequence fragments [7]), the growth of experimental protein structures [8] and predicted structures remains to catch up (the AlphaFold database contains over 200 million predicted structures [9]). Such sequences-structures gap has motivated the development of new computational tools that make functional sense of protein sequences without directly using structural information (for example, by using deep learning to train large protein language models [10]). Recently, AlphaMissense attained state of the art prediction of missense variant pathogenicity by adapting AlphaFold fine-tuned on human and primate variant population frequency databases [11].

A major interest in personalized medicine is in understanding novel genetic variations through genotype-phenotype association studies in relation to diseases. Particularly, a rapidly growing number of non-synonymous single nucleotide variants (nSNVs) have been uncovered in protein coding regions that can adversely impact protein function and cause diseases [12]. Various computational methods were developed using evolutionary conservation and phylogeny in combination with biochemical and structural properties of amino acids to discriminate neutral vs. deleterious nSNVs [13–22]. Protein structural dynamics has also proven useful in discovering functionally important residues [23, 24] which could constitute hot spots for disease-causing nSNVs [25, 26]. However, the requirement of 3D structures has limited the number of nSNVs that can be analyzed by existing structure-based computational tools, although such constraint has been significantly alleviated by recent progress in protein structure prediction [27].

As alternatives to structure-based methods, sequences-based coevolution analysis has become increasingly powerful in predicting structural couplings between pairs of contacting residues [28–31], thanks to the development of direct coupling methods that can overcome the confounding indirect coupling effects [29, 32, 33]. In principle, coevolving pairs of residues can be identified from a sufficiently large multiple sequence alignment, allowing the prediction of close spatial proximity in the native structures. Boosted by deep learning and other algorithmic developments, this coevolution analysis has led to accurate prediction of residue contacts which make *de novo* protein structure prediction possible [28]. Furthermore, coevolution analysis (enhanced by deep learning) has also been used to study various aspects of protein functional interactions such as allostery [34]. For example, RaptorX uses an ultra-deep neural network combining coevolution information with sequence conservation information to infer 3D contacts with higher accuracy than previous methods [35, 36]. DeepMetaPSICOV [37] combines the input feature sets used by earlier methods (MetaPSICOV [38] and DeepCov [39]) as input to a deep, fully convolutional residual neural network. SPOT-Contact predicts protein contact maps by stacking residual convolutional networks with two-dimensional residual bidirectional recurrent LSTM networks, and using both one-dimensional sequence-based and two-dimensional evolutionary coupling based information [40]. These three state-of-the-art coevolution analysis methods are employed in this study to construct protein residue contact maps for network analysis (see below).

Another line of protein research is based on the treatment of a protein as a network where amino acid residues are nodes and their bonded/non-bonded interactions form edges [41]. Such models can be readily built upon 3D native structures so that a whole suite of network analysis tools (see https://networkx.org/) can be applied. For example, Amitai et al [42] used network analysis of protein structures (using closeness centrality) to identify functional residues. Going beyond network analysis, deep-learning-based study of protein graph neural networks is an active area of research [43].

In a recent paper, Butler et al [44] proposed a sequence-based Gaussian network model (Seq-GNM) to calculate the dynamic profile of a protein without a 3D structure. They used coevolution analysis to build a network model which connects residues predicted to be in contact via evolutionary couplings. Their work built on previous studies that shown crystallographic B-factors are useful in predicting the impact of nSNVs on protein function [45, 46]: rigid sites with low B-factors are more susceptible to destabilizing nSNVs than flexible sites with high B-factors. Indeed, existing computational tools to diagnose neutral and deleterious nSNVs (such as PolyPhen-2 [47]) use crystallographic B-factors along with other evolutionary and structural features. More specifically, Butler et al used Seq-GNM to compute B-factors for protein residues, and they found that deleterious nSNVs are overabundant at low B-factor sites, while neutral nSNVs are overabundant at high B-factor sites. Mechanistically, low B-factors may indicate that a site is crucial for maintaining structural stability and/or modulating functional motions (as a hinge) and thus susceptible to mutations. In contrast, high B-factors are associated with flexible regions with minimal interactions, which are thus more robust to mutations. Based on these observations, they proposed that the sequences-based predicted B-factors can discriminate between deleterious and neutral nSNVs without structural information.

Inspired by the above study and recent progress in machine learning, network analysis, and protein language models, we further develop the sequences-based protein residue network analysis in the following directions: 1. We build protein residue networks using three different coevolution analysis tools (RaptorX, DeepMetaPSICOV, and SPOT-Contact) as enabled by deep learning. 2. We exploit three machine learning algorithms (Random Forest, Gradient Boosting, and Extreme Gradient Boosting) to optimally combine 20 distinct network node centrality scores as calculated from the contact probability matrices to predict hot spot residues for disease mutations. 3. Based on a dataset of 107 proteins with known deleterious/neutral mutations, we evaluate our sequences-based network scores both individually and in combination, and then compare with alternative structures-based network scores and a physics force field based method. By optimally combing three coevolution analysis methods and the resulting 20 network scores by machine learning, we are able to discriminate deleterious and neutral mutation sites accurately (AUC of ROC ~ 0.84), which is on par with structure-based network scores (AUC ~ 0.83). Furthermore, by combining our method with a state-of-the-art predictor of the functional effects of sequence variation based on large protein language models (ESM [48]), we have significantly improved the prediction of disease variant sites (AUC ~ 0.89).

In the following sections, we first describe the detailed methodology in the order of the proposed workflow, then we report the results of evaluation of our network-based scores both individually and collectively (via machine learning), finally we discuss specific case studies of four proteins to illustrate the usage of our method.

## Materials and methods

Here is a summary of the workflow of our coevolution-based method for predicting key variant sites:

- Collect datasets of protein sequences and variants (see Section 1)
- Run co-evolution analysis of a given target protein sequence to build a residue contact map P (see Section 2)
- Use NetworkX to calculate node centrality scores based on P (see Section 3)
- Use sequence-based GNM to calculate additional node importance scores (see Section 4)
- (optional) Use protein language model (ESM) to predict variant importance (see Section 5)
- (optional) Use AlphaFold and FoldX to predict variant importance (see Section 6 and 7)
- Use machine learning to optimally combine the above scores for classifying deleterious vs neutral variant sites (see Section 8)

### 1. Datasets of protein sequences and variants

A dataset of 107 protein sequences with ≤500 residues and ≥20 annotated deleterious/neutral variants were collected from the HumVar database [47] (sources: humvar-2011_12.deleterious.pph.input and humvar-2011_12.neutral.pph.input from ftp://genetics.bwh.harvard.edu/pph2/training/training-2.2.2.tar.gz). Their UniProt ids and sequences can be accessed at https://simtk.org/projects/hotspots. This diverse dataset contains 97 proteins with their pairwise sequence identity < 30%.

The HumVar dataset consists of 13,032 human disease-causing mutations from UniProt and 8,946 human nonsynonymous single-nucleotide polymorphisms (nsSNPs) without annotated involvement in disease. This dataset was previously used to train and test PolyPhen-2 [47] for predicting damaging effects of missense mutations, and was used by Butler et al [44] in benchmarking their seq-GNM method for predicting deleterious/neutral nSNVs.

Since this dataset is highly imbalanced (there are 4040 deleterious mutation sites but only 120 neutral mutation sites) [49], we have added 3403 additional neutral sites with very low conservation scores (i.e. grade ≤2 as assessed by the ConSurf program [50]). Our objective is to train and test a binary classifier of residues in these proteins as deleterious or neutral. To this end, we split 107 proteins into training and testing sets (with 79 and 28 proteins, respectively), and perform evaluations based on the testing set. The main metric of evaluation is the ROC curves and associated area under the curve (AUC). AUC is a standard metric for evaluating binary classifiers based on the ROC curve of sensitivity and specificity. The ROC curves are also used in other computational papers for variant prediction (see [47]).

### 2. Sequences-based coevolution analysis and protein contact map construction

We perform coevolution analysis using three state-of-the-art methods: the RaptorX server (http://raptorx.uchicago.edu), the DeepMetaPSICOV server (http://bioinf.cs.ucl.ac.uk/psipred/), and the SPOT-Contact server (https://sparks-lab.org/server/spot-contact/). A sequence length limit (500) is imposed by the capacity of coevolution analysis servers, and may be circumvented if installing and running coevolution analysis locally.

These methods use multiple sequence alignments to compute the probability P_ij_ of residue pair (i, j) forming spatial contact. Based on the matrix of predicted P_ij_, a protein residue contact map can be built with residues as nodes and pairwise contacts as edges weighted by P_ij_. By default, we do not apply any threshold cutoff to P_ij_ for defining contacts (unless networks with unweighted edges are required by some node centrality algorithms in NetworkX, where we remove edges with P_ij_<0.1, and set weight to 1 for the remaining edges).

### 3. Network analysis of protein contact map

By treating a protein contact map as a network of nodes and edges, we calculate various node centrality scores to predict key residues as hotspots for disease mutations.

A simple score to measure node centrality is a weighted node degree that accounts for the nearest neighbor interactions (denoted *W*_*1*_):

$$W_{1,i}=\underset{k\neq i}{\sum }P_{ik}$$ (1)

To include indirect couplings beyond the nearest neighbors, we calculate the node degree based on the n’th power of the contact probability matrix (denoted *W*_*n*_):

$$W_{n,i}=\underset{k\neq i}{\sum }P_{ik}W_{n-1,k}=\underset{k\neq i}{\sum }{P^{n}}_{ik}$$ (2)

As n goes to infinity, *W*_*n*_ converges to the eigenvector of P matrix with the highest eigenvalue *λ*_max_ (denoted *W*_∞_):

$$PW_{\infty }=\lambda_{\max}W_{\infty }$$ (3)

Among various *W*_*n*_, *W*_*2*_ can be interpreted as the node degrees of a new network based on a neighborhood similarity matrix *S* as follows (denoted *W*_*s*_):

$$S_{ij}=\underset{k\neq i,j}{\sum }P_{ik}P_{jk},\;W_{s,i}=\underset{k\neq i}{\sum }S_{ik}$$ (4)

In this study we use five network scores (*W*_*1*_, *W*_*2*_, *W*_*3*_, *W*_*∞*_ and *W*_*s*_) as predictive features for node importance. Additionally, we exploit 13 network centrality metrics as calculated by the NetworkX package (see Table 1). To allow meaningful comparison of scores between proteins, the scores of each protein are sorted and their ranking percentiles are linearly transformed to values between 0 and 1.

**Table 1:** Network centrality scores as implemented in the NetworkX package. (see https://networkx.org/documentation/stable/reference/algorithms/centrality.html).

Symbol	Centrality name	Definition
C1	degree_centrality	Corresponding to W_1_
C2	eigenvector_centrality	Corresponding to W_∞_
C3	closeness_centrality	Closeness centrality of a node u is the reciprocal of the average shortest path distance to u over all n-1 reachable nodes.
C4	betweenness_centrality	Betweenness centrality of a node u is the sum of the fraction of all-pairs shortest paths that pass through u.
C5	current_flow_closeness_centrality	Current-flow closeness centrality is a variant of closeness centrality based on effective resistance between nodes in a network.
C6	current_flow_betweenness_centrality	Current-flow betweenness centrality is based on an electrical current model for information spreading.
C7	communicability_betweenness_centrality	Communicability betweenness centrality is based on the number of walks connecting every pair of nodes.
C8	load_centrality	Load centrality of a node u is the fraction of all shortest paths that pass through u.
C9	subgraph_centrality	Subgraph centrality of a node u is the sum of weighted closed walks of all lengths starting and ending at u.
C10	harmonic_centrality	Harmonic centrality of a node u is the sum of the reciprocal of the shortest path distances from all other nodes to u.
C11	second_order_centrality	Second order centrality of a node u is the standard deviation of the return times to u of a perpetual random walk on G.
C12	laplacian_centrality	Laplacian Centrality of a node u is measured by the drop in the Laplacian Energy after deleting u from the graph.
C13	katz_centrality_numpy	Katz centrality computes the centrality for a node u based on the centrality of its neighbors. It is a generalization of the eigenvector centrality.

### 4. Sequences-based GNM

For comparison, we implemented Bulter et al’s sequence-based GNM [44]. The original structure-based Gaussian network model (GNM) represents a protein structure as an elastically connected network of residues to obtain the equilibrium fluctuations of residues. In the absence of a structure, the sequence-based GNM (Seq-GNM) treats coevolving residue pairs as contacting pairs.

To construct the Kirchhoff matrix (denoted *K*), each non-bonded residue pair is assigned a value of -1 times its contact probability. The bonded residue pairs (i, i+1) are assigned -1 to enforce local chain connectivity. The diagonal elements of *K* are assigned so that the sum of each row and column is zero:

$$K_{ij}=\{\begin{array}{cc} -P_{ij} & i\neq j\\ \sum_{k\neq i}P_{ik} & i=j \end{array}$$ (5)

The vibrational thermal fluctuations of residues are evaluated by inverting the Kirchhoff matrix (or summing over the modes as weighted by 1/λ_m_). The per-residue mean-square fluctuations (MSF), which are proportional to the crystallographic B factors, are given as follows:

$$MSF_{i}\propto K_{ii}^{-1}=\underset{m>0}{\sum }\frac{{V_{mi}}^{2}}{\lambda_{m}}$$ (6)

where the eigen-decomposition of *K* gives eigenvectors *V*_*m*_ and eigenvalues λ_m_ that satisfy:

$$KV_{m}=\lambda_{m}V_{m}$$ (7)

Low-MSF residues correspond to rigid cores or hinges of dynamical importance [44].

As an alternative way to evaluate node importance using GNM, we perform a perturbation-based hotspot analysis as follows: For mode *m*, calculate how much its eigenvalue changes (*δλ*_*m*,*i*_) in response to a perturbation at a chosen residue position i [23, 24, 51] (i.e., by uniformly weakening the contacts with residue i). Then compute $\delta \lambda_{i}=\sum_{m}\delta \lambda_{m,i}$ to assess the dynamic importance of this residue position [52]. High-*δλ*_*i*_ residues correspond to sites highly sensitive to local perturbations that mimic mutations.

The above two GNM-based scores are combined with the other network scores for machine learning.

### 5. ESM based variant prediction

For comparison with our method, we use a deep-learning variant predictor based on a large protein language model (ESM). We downloaded and installed the ESM package and pretrained models from https://github.com/facebookresearch/esm. Since our dataset consists of known variants (from HumVar) and added non-conserved sites (with specific mutations unknown), we simulate the mutational effects on each site by introducing Alanine substitution if the wildtype residue is not an Alanine and Glycine substitution otherwise [53]. Then we process the mutated sequence with 5 pretrained ESM models (esm1v_t33_650M_UR90S_1, esm1v_t33_650M_UR90S_2, esm1v_t33_650M_UR90S_3, esm1v_t33_650M_UR90S_4, and esm1v_t33_650M_UR90S_5), which predict the difference in the probability of observing the widetype residue and the mutant residue at a given site [48]. We record the predictions of five ESM models as separate features to be optimally integrated via machine learning.

### 6. AlphaFold for structural prediction

We downloaded predicted structures for the 107 proteins from AlphaFold DB (https://alphafold.ebi.ac.uk/). A residue contact probability matrix is constructed based on the predicted structures as follows:

$$P_{ij}=\frac{1}{1+e^{d_{ij}-10}}$$ (8)

where *d*_*ij*_ is the distance between residues i and j, and 10 Å is used as a soft cutoff distance. We then use this contact probability matrix to perform the same network analysis as in the sequences-based method and for optimization with machine learning.

### 7. FoldX for structural refinement and Alanine scanning analysis

FoldX program [54] was downloaded from https://foldxsuite.crg.eu/. We use the RepairPDB command to refine the AlphaFold-predicted models (by fixing bad torsion angles and Van der Waals clashes). Then we use the AlaScan command to mutate each residue to Ala and calculate the resulting changes in Gibbs free energies which are then used as a feature to predict hotspots of disease mutations.

### 8. Machine learning algorithms

We use the following machine learning methods of the scikit-learn package (https://scikit-learn.org/stable/) to learn optimal combinations of multiple features (see Table 3) to predict if a given site is deleterious or neutral mutation site:

Random Forest Classifier (RF) (sklearn.ensemble.RandomForestClassifier): A random forest is a meta estimator that fits a number of decision tree classifiers on various sub-samples of the dataset and uses averaging to improve the predictive accuracy and control over-fitting. We tune the following hyper-parameters: max_depth, n_estimators, max_features.

Gradient Boosting Classifier (GB) (sklearn.ensemble.GradientBoostingClassifier): This algorithm builds an additive model in a forward stage-wise fashion. In each stage a regression tree is fit on the negative gradient of the loss function, e.g. binary log loss. We tune the following hyper-parameters: n_estimators, max_depth, max_features.

Extreme Gradient Boosting Classifier (XGB) (xgboost.XGBClassifier): This algorithm is an optimized distributed version of gradient boosting designed to be highly efficient, flexible and portable. We tune the following hyper-parameters: n_estimators, max_depth, reg_alpha, reg_lambda.

The above three methods were chosen because they have performed successfully in machine learning contests in Kaggle (see https://www.packtpub.com/product/the-kaggle-book/9781801817479). They are also relatively cheap to train and optimize compared with the more computationally intensive deep learning methods.

We use Optuna (https://optuna.org/) for hyper-parameter tuning of the above algorithms. We have run Optuna multiple times to ensure the resulting best metric is reproducible.

## Results and discussion

This study explores how to systematically utilize the coevolution information from multiple sequence alignments to model and analyze a protein as a residue contact network beyond the scope of GNM. To this end, we first use coevolution analysis to construct a protein residue contact map with edges weighted by the predicted contact probability; then we exploit an array of 20 network-based scores to assess the node importance as predictors for disease mutation sites; finally we evaluate the predictive power of these scores individually and collectively (using machine learning) based on a subset of 107 protein sequences and their variants from the HumVar database. For comparison, we also evaluate alternative methods based on AlphaFold predicted protein structures, a physics-based force field (FoldX), and a protein language model (ESM).

### 1. Evaluation of individual network scores

Based on the protein residue contact maps built from three coevolution analysis tools (DeepMetaPSICOV, RaptorX, and SPOT-Contact), we applied network analysis to calculate 20 network scores (see Table 2), measuring node centrality using various different algorithms as implemented in NetworkX or based on seq-GNM (see Methods). These scores include simple weighted node degrees for n-hop nearest neighbors (see Methods) and more sophisticated centrality metrics (see Table 1), along with 2 seq-GNM based scores that probe network dynamics (MSF and δλ, see Methods). The use of a wide variety of scores aims to fully capture the node importance at different levels. We evaluate the performance of each score using the AUC of ROC for the testing set, which provides a balanced evaluation of sensitivity and specificity (see Table 2) without arbitrarily fixing the cutoff. More specifically, we sort all testing-set variants by a particular score and predict a variant deleterious/neutral if its score is above/below a sliding cutoff value. This results in an ROC curve for which one can calculate the AUC (see Table 2).

**Table 2:** Evaluation of 20 network scores based on protein residue contact maps constructed from 3 coevolution analysis tools (DeepMetaPSICOV, RaptorX, and SPOT-Contact) and AlphaFold-predicted structures.

Score	AUC* of	AUC* of	AUC* of	AUC* of
	DeepMetaPSICOV	RaptorX	SPOT-Contact	AlphaFold
C1	0.74	0.76	0.73	0.82
C2	0.73	0.74	0.76	0.77
C3	0.76	0.73	0.69	0.73
C4	0.64	0.54	0.60	0.58
C5	0.78	0.76	0.79	0.80
C6	0.63	0.58	0.67	0.60
C7	0.75	0.61	0.72	0.74
C8	0.64	0.54	0.60	0.58
C9	0.77	0.76	0.74	0.78
C10	0.75	0.73	0.68	0.75
C11	0.79	0.76	0.77	0.80
C12	0.77	0.77	0.79	0.83
C13	0.73	0.73	0.76	0.76
δλ	0.79	0.76	0.78	0.83
MSF	0.79	0.76	0.78	0.80
W_1_	0.79	0.77	0.78	**0.83**
W_2_	0.80	0.78	0.80	0.83
W_3_	**0.80**	**0.78**	**0.81**	0.82
W_∞_	0.80	0.74	0.79	0.77
W_s_	0.80	0.78	0.80	0.83
FoldX				0.68

* The AUC is calculated based on the ROC for all variants of the 28 testing set proteins. Alternatively, we also calculated AUCs based on the ROCs of individual proteins and their summary statistics (see S1 Table).

Overall, DeepMetaPSICOV (max AUC = 0.80) and SPOT-Contact (max AUC = 0.81) perform slightly better than RaptorX (max AUC = 0.78), which may be attributed to their use of different deep learning architectures with different expressiveness. Interestingly, simple weighted node degrees (W_1_, W_2_, and W_3_) perform better than those more complex centrality scores (see Table 2). When computing node degrees, going beyond the nearest neighbors seems to improve the prediction slightly (see Table 2). Two GNM-based scores perform similarly but slightly worse than the weighted node degrees (see Table 2). Among those NetworkX-based scores (see Table 1), C5, C11 and C12 outperform the others, while those betweenness-based scores (C4, C6, and C8) underperform (see Table 2). Taken together, the above findings suggest that the functional importance of a node/residue is better captured by its role as a highly-connected hub (with high degrees) than alternative mechanisms (e.g. a key role in dynamics or an information bottleneck of the shortest paths, etc). That said, we cannot discount possible contributions to the functional importance by more subtle and complex mechanisms which may involve a subnet or pathway of multiple residues.

For comparison with alternative methods, we evaluated the performance of variant effect prediction by five pre-trained protein language models (ESM, see Methods), and the resulting AUC varies between 0.79 and 0.81, which are comparable to our coevolution-based network scores (see Table 2). This is encouraging given that the ESM-based zero-shot prediction of variant effect was shown to perform at state of the art [48]. For further comparison of our sequences-based methods with structures-based methods, we also performed network analysis based on protein structures as predicted by AlphaFold (see Methods). Overall, the structures-based scores (max AUC = 0.83) perform slightly better than the sequences-based scores. This may be partly due to the structure-based contact maps (see Eq 8) being more sharply defined than the fuzzier/noisier contact-probability-based contact maps. Notably, when structures are used, the network-based scores perform significantly better than a physics-based force field (FoldX, see Methods) with AUC = 0.68. Taken together, these findings support the usefulness of individual sequences-based network centrality scores in predicting important residues in comparison with alternative more sophisticated methods.

To further understand the different accuracies of the above scores, we explore the relationships between them by evaluating the pairwise Pearson correlations (PC) (see S2 Table). W_1_, W_2_, W_3_, W_∞_, W_s_, MSF and δλ are highly correlated (with PC≥0.93 for DeepMetaPSICOV, PC≥0.84 for SPOT-Contact, PC≥0.86 for AlphaFold), although their correlations are somewhat weaker for RaptorX. Among the NetworkX-based scores (see Table 1), C5, C11 and C12 are also highly correlated with the above scores. Such strong correlations suggest that these scores have all captured the same essential features (i.e. high local connectivity) predictive of their importance, which explain their high AUC (see Table 2). In contrast, the betweenness-based scores (C4, C6, and C8) do not correlate well with the above scores, and they are worse predictors of node importance as indicated by their low AUC (see Table 2). The overall high correlations among those better-performing scores (with high AUC) imply their combinations may not lead to substantial improvement in performance (see below).

In summary, by evaluating 20 network scores individually, we have found a wide range of performance with AUC varying from 0.54 to 0.81 (see Table 2). The top-performing scores seem to correlate strongly with each other, so they must have captured a common aspect of node centrality that is relevant to functional importance (e.g. high local connectivity instead of high betweenness). Interestingly, the two GNM-based scores, despite measuring distinct dynamic properties (MSF measures thermal fluctuations while δλ measures sensitivity to local perturbations), are also strongly correlated with each other and those degree-based network scores. Therefore, to speed up the variant prediction workflow we only need to compute those simpler weighted node degrees as features without significantly losing accuracy.

### 2. Combining all network scores to predict variant hotspots by machine learning

To optimize the predictive power of the above network scores based on three coevolution analysis methods (and optionally AlphaFold and ESM), we have employed machine learning algorithms (see Methods) to take them as input features, train a binary classifier which predicts if a residue position is linked to neutral or deleterious variants (using first 79 proteins as training set), and then test its prediction using the remaining 28 proteins as testing set. We use the AUC of ROC as the metric for assessing the prediction quality of the trained classifier. Various schemes of combining features from different methods have been explored and compared (S1-S9, see Table 3 for details).

**Table 3:** Evaluation of classifiers trained by 3 machine learning algorithms (RF, GB and XGB, see Methods) by combining scores derived from 3 coevolution analysis tools (DeepMetaPSICOV, RaptorX, and SPOT-Contact), AlphaFold-predicted structures, and protein language models (ESM). Total 9 schemes for combining features were studied (S1—S9).

schemes of combining features	AUC of RF	AUC of GB	AUC of XGB
S1:20 scores (see Table 2) from DeepMetaPSICOV	0.81	0.81	0.81
S2:20 scores (see Table 2) from RaptorX	0.80	0.80	0.80
S3:20 scores (see Table 2) from SPOT-Contact	0.82	0.82	0.82
S4:20 scores (see Table 2) from AlphaFold	0.83	0.83	0.83
S5:5 scores from ESM (see Methods)	0.83	0.83	0.83
S6:3×20 scores from all three coevolution methods	0.84	0.84	0.84
S7:3×7 scores from all three coevolution methods (w/o C1-C13)	0.82	0.82	0.83
S8:3×20+5 scores from all coevolution methods and ESM	0.89	0.89	0.89
S9:20+5 scores from AlphaFold and ESM	0.88	0.88	0.88

To evaluate the protein residue contact maps constructed by each method, we combine all 20 network scores (see Table 2) based on the contact maps predicted by the same method for machine learning. The resulting AUC for each coevolution analysis method (DeepMetaPSICOV, RaptorX, and SPOT-Contact) is 0.81, 0.80, and 0.82, respectively (see S1, S2 and S3 in Table 3), which are slightly better than the best AUC of individual scores (0.78~0.81, see Table 2). This lack of substantial improvement may be attributed to high correlations among the top-performing scores (see S2 Table) which limit the effectiveness of ensemble learning. For comparison, we also trained and tested classifiers using the AlphaFold-predicted structures-based contact maps, and alternative classifiers based on a protein language model ESM (see S4 and S5 in Table 3). Both alternative methods give comparable yet slightly better AUC (0.83). Similar to our finding, Butler et al reported AUC of 0.81 after combining the B-factors of Seq-GNM with evolutionary features, but using a different dataset [44].

To further boost the prediction performance, we have sought to combine the network scores of all three coevolution analysis methods for machine learning (see S6 in Table 3), resulting in a better AUC (0.84) which slightly outperforms both AlphaFold and ESM (0.83). To assess the added value of including 13 NetworkX-based centrality scores (see Table 1), we have performed an ablation study that excludes them in machine learning (see S7 in Table 3), and found slightly lower AUC (0.82~0.83). By not using the NetworkX-based centrality scores, one can speed up the calculation without significantly reducing performance. Taken together, our findings support the power of combining an array of different network scores from different coevolution analysis tools to optimize the prediction in the framework of ensemble learning, where multiple distinct predictive methods complement each other and yield better and more stable performance than any single method.

To further explore how well our coevolution-based method complements alternative methods, we have combined all the network scores with the ESM scores in machine learning (see S8 in Table 3). Encouragingly, we have obtained markedly improved AUC (0.89), which is comparable to machine learning that combines the AlphaFold-predicted structures-based network scores with the ESM scores (see S9 in Table 3, AUC = 0.88). Therefore, our method is highly complementary to the state-of-art ESM in variant effect prediction

For comparison with other studies, Butler et al showed that Seq-GNM combined with evolutionary parameters attained a sensitivity of 0.84 and a specificity of 0.66 [44]. PolyPhen-2 achieved a sensitivity of 0.73 and a specificity of 0.8 on the HumVar datasets [47]. While using different training and testing datasets, we have attained competitive results with a sensitivity of 0.82 and a specificity of 0.80 (using all the network scores from three coevolution analysis tools and the ESM scores). For more direct comparison, we also evaluated PolyPhen-2 based on the same 28 testing-set proteins and their variants, and obtained an AUC of 0.85, which is close to our method (see Table 3). However, this metric is likely positively biased since PolyPhen-2 has been trained on the HumVar dataset.

In summary, via extensive machine learning, we have demonstrated the power of using an ensemble of sequences-based network scores calculated by different co-evolution analysis tools to accurately predict deleterious mutation sites. Although some network scores are highly correlated (see S2 Table) and they vary widely in accuracy (see Table 2), these scores are shown to be very informative in enabling effective ensemble learning when combined with alternative methods like ESM.

### 3. Case studies

To illustrate the biomedical significance of our predictions of variant sites with network scores, we discuss in details the following four proteins from our dataset. To help visualize our detailed predictions in comparison with the ‘ground truth’, we color residue positions by the network scores (i.e. red/blue for high/low score), and show known deleterious and neutral variant sites as large and small balls, respectively. In such a visual scheme, a large red ball indicates a true positive prediction, a small blue ball indicates a true negative prediction, and so on.

#### Glucose-6-phosphate exchanger

(Uniprot id: O43826): As an inorganic phosphate and glucose-6-phosphate antiporter, it transports cytoplasmic glucose-6-phosphate into the lumen of the endoplasmic reticulum and translocates inorganic phosphate in the opposite direction. Being involved in glucose production through glycogenolysis and gluconeogenesis, it plays a central role in homeostatic regulation of blood glucose levels. It is linked to diseases like congenital disorder of glycosylation and glycogen storage disease (see https://www.uniprot.org/uniprotkb/O43826/entry#function).

**Fig 1:**
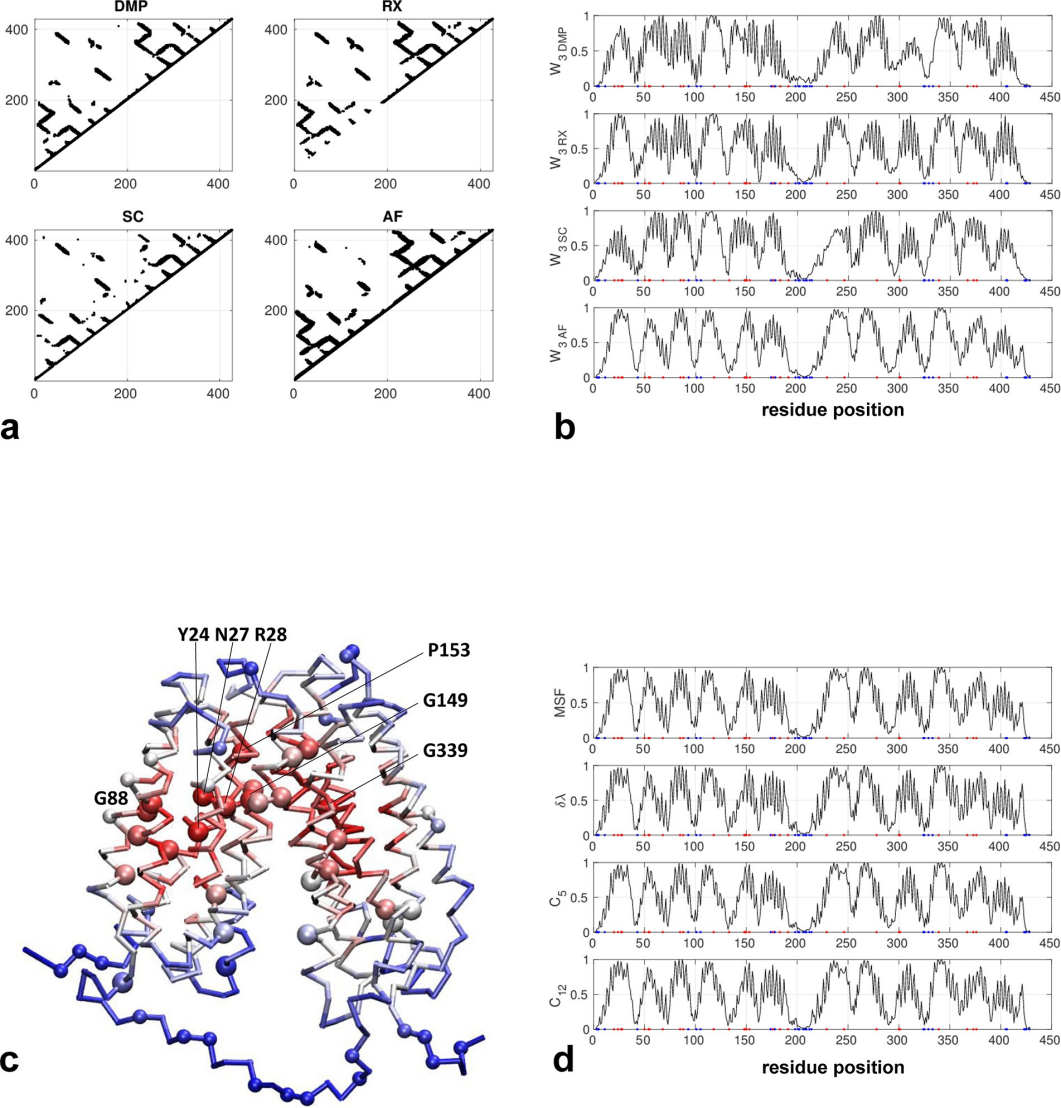
Results for glucose-6-phosphate exchanger (Uniprot id: O43826). (a) Four contact maps constructed from coevolution analysis by DeepMetaPSICOV (DMP), RaptorX (RX), SPOT-Contact (SC), and the predicted structure by AlphaFold (AF) (only those contacts with probability >0.1 are shown). (b) W_3_ scores for all residue positions based on the contact maps in (a), where red and blue dots mark residues with deleterious and neutral mutations, respectively. (c) Predicted structure by AlphaFold as colored by W_3_ scores (red/blue for high/low values), where residues with deleterious and neutral mutations are shown as large and small balls, respectively (G20, Y24, N27, R28, G50, S54, S55, G68, L85, G88, W118, Q133, A148, G149, G150, P153, C176, C183, P191, L229, W246, I278, R300, H301, G339, A367, A373, G376, see https://www.uniprot.org/uniprotkb/O43826/variant-viewer). (d) Four other network scores (MSF, δλ, C5 and C12) for all residue positions based on the contact maps in (a).

The AlphaFold-predicted structure forms a dimer of transmembrane helical domains with most deleterious mutation sites concentrated inside the central core while those non-conserved residues (i.e. neutral mutation sites) are mostly located on the periphery (see Fig 1C). The contact maps predicted by three coevolution analysis tools all agree well with the contact map based on the AlphaFold structure (see Fig 1A) (except that RaptorX omitted many local contacts in residues 1–200). As a result, the network centrality scores (W_3_) also agree well between these methods (see Fig 1B), although the coevolution-based network scores are generally noisier (with more spikes) than the structure-based scores (see Fig 1B). Different network scores calculated from the same contact map are also highly similar (see Fig 1D) despite being based on different algorithms. For example, scores of δλ and MSF agree very well (see Fig 1D). Encouragingly, those residues identified with high network scores are primarily within the central core (inside each domain or in the inter-domain hinge region), thus overlapping with most deleterious mutations (see a cluster of large red balls in Fig 1C). Among those top-10% predicted hotspot residues (see Fig 1C), mutations Y24H, N27K, R28H, G88D, G149E, P153L, and G339C were implicated in causing glycogen storage disease [55]. Two of these mutations (R28H and G149E) were found to exhibit undetectable microsomal glucose-6-phosphate transport activity in transient expression studies [56], thus confirming their functional importance.

#### Presenilin-1

(Uniprot id: P49768): As the catalytic subunit of the gamma-secretase complex, it catalyzes the intramembrane cleavage of integral membrane proteins such as Notch receptors. It is involved in various diseases including a familial early-onset form of Alzheimer disease and a form of frontotemporal dementia (see https://www.uniprot.org/uniprotkb/ P49768/entry#function).

**Fig 2:**
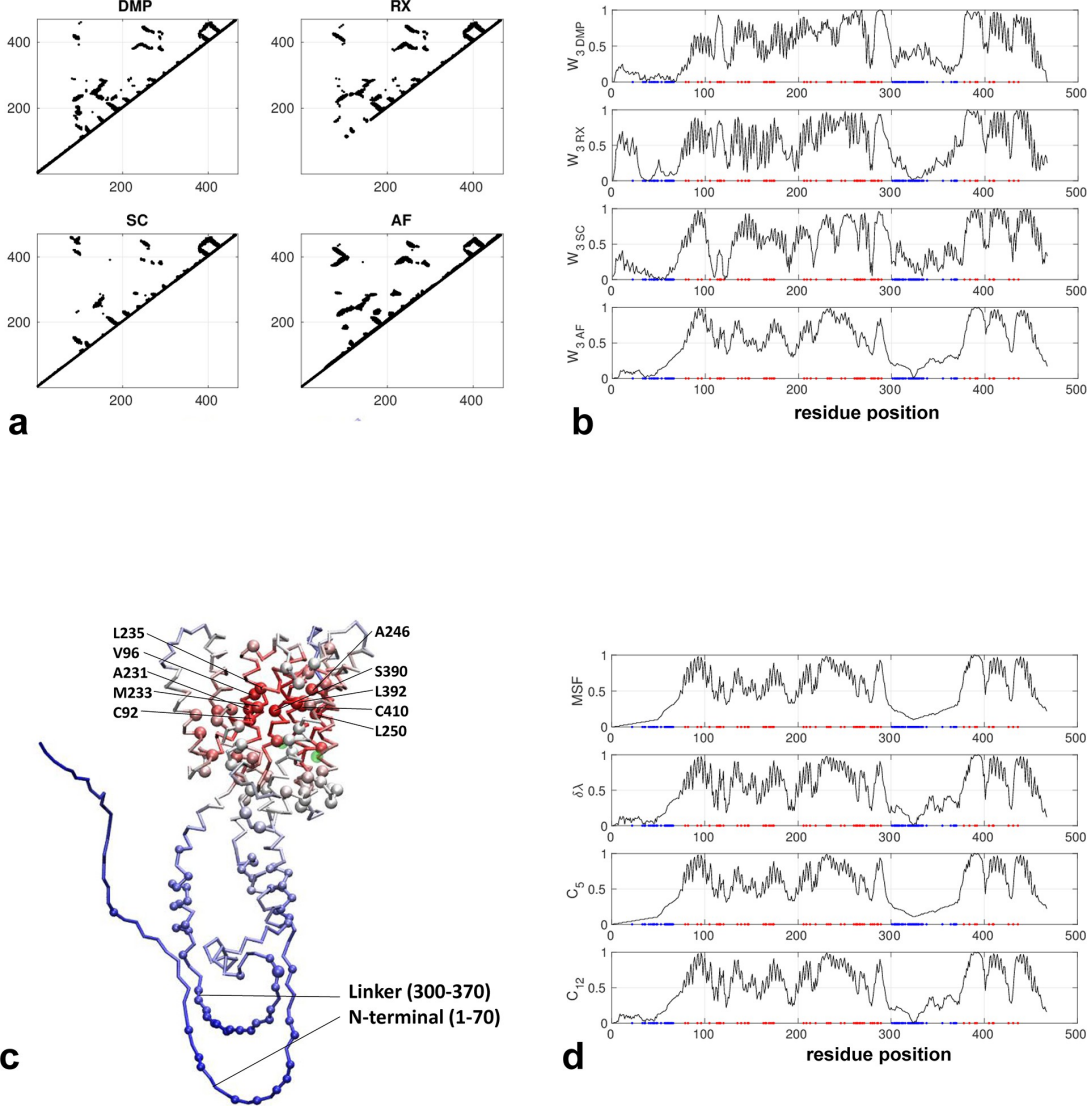
Results for presenilin-1 (Uniprot id: P49768). (a) Four contact maps constructed from coevolution analysis by DeepMetaPSICOV (DMP), RaptorX (RX), SPOT-Contact (SC), and the predicted structure by AlphaFold (AF) (only those contacts with probability >0.1 are shown). (b) W_3_ scores for all residue positions based on the contact maps in (a), where red and blue dots mark residues with deleterious and neutral mutations, respectively. (c) Predicted structure by AlphaFold as colored by W_3_ scores (red/blue for high/low values), where residues with deleterious and neutral mutations are shown as large and small balls, respectively (A79, V82, C92, V96, F105, L113, Y115, T116, P117, E120, N135, M139, I143, M146, T147, H163, W165, L166, S169, L171, L173, L174, G206, G209, I213, L219, A231, M233, L235, A246, L250, A260, L262, C263, P264, G266, P267, R269, L271, R278, E280, L282, A285, L286, S289, D333, G378, G384, S390, L392, N405, A409, C410, A426, A431, P436, see https://www.uniprot.org/uniprotkb/ P49768/variant-viewer), and active-site residues are colored in green. (d) Four other network scores (MSF, δλ, C5 and C12) for all residue positions based on the contact maps in (a).

The AlphaFold-predicted structure consists of two closely packed helical domains with most deleterious mutations clustered inside the core domain while the non-conserved residues are mostly located on the N-terminal loop (residues 1–70) and the inter-domain linker (residues 300–370) (see Fig 2C). The active site [57] (D257 and D385) is also located in the core domain (colored green in Fig 2C). The contact maps predicted by three coevolution analysis methods all resemble the contact map based on the predicted structure (see Fig 2A) (except that RaptorX omitted local contacts in residues 1–100). As a result, the network scores agree well between them in the helical domains (see Fig 2C), but with more differences in the flexible regions (residues 1–70 and 300–370). Reassuringly, those residues identified by high network scores are primarily clustered within the central core overlapping with most deleterious mutations (see a cluster of large red balls in Fig 2C), while the flexible N-terminal and linker feature low scores consistent with low sequence conservation (see a cluster of small blue balls in Fig 2C). Among those top 10% predicted hotspot residues (see Fig 2C), mutations at C92, V96, A231, M233, L235, A246, L250, S390, L392, and C410 were found to cause loss of function and altered amyloid-beta production [58]: C92S led to loss of protease function and increased Abeta42 levels. V96F caused loss of protease activity. A231T/V and M233T led to decreased protease activity, altered amyloid-beta production and increased amyloid-beta 42/amyloid-beta 40 ratio. L235P/R and S390I abolished protease activity. A246E and L250S abolished protease activity and increased amyloid-beta 42/amyloid-beta 40 ratio. L392V resulted in reduced proteolysis, altered amyloid-beta production and increased amyloid-beta 42/amyloid-beta 40 ratio. C410I reduced proteolysis. Since most of these residues are not near the active site, their effects on protease activity are likely allosteric.

#### b(0,+)-type amino acid transporter 1

(Uniprot id: P82251): It forms a functional transporter complex that mediates the electrogenic exchange between cationic amino acids and neutral amino acids. Its dysfunction is linked to Cystinuria, an autosomal disorder characterized by impaired epithelial cell transport of cystine and dibasic amino acids in the proximal renal tubule and gastrointestinal tract (see https://www.uniprot.org/uniprotkb/P82251/entry#function).

**Fig 3:**
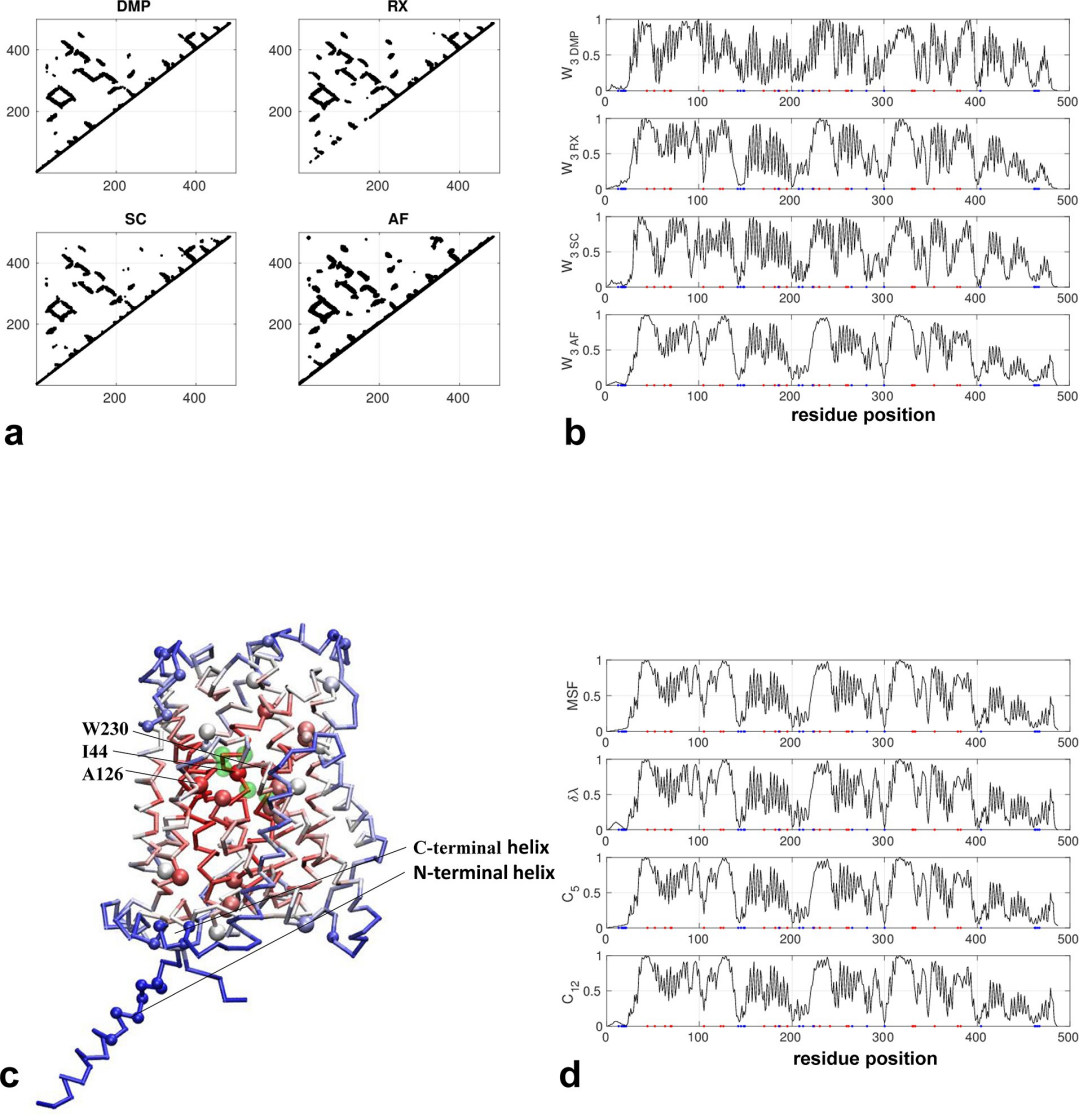
Results for amino acid transporter 1 (Uniprot id: P82251). (a) Four contact maps constructed from coevolution analysis by DeepMetaPSICOV (DMP), RaptorX (RX), SPOT-Contact (SC), and the predicted structure by AlphaFold (AF) (only those contacts with probability >0.1 are shown). (b) W_3_ scores for all residue positions based on the contact maps in (a), where red and blue dots mark residues with deleterious and neutral mutations, respectively. (c) Predicted structure by AlphaFold as colored by W_3_ scores (red/blue for high/low values), where residues with deleterious and neutral mutations are shown as large and small balls, respectively (V142,L223,I44,P52,G63,W69,A70,G105,T123,A126,V170,A182,I187,G195, A224,W230,I241,G259,P261,V330,A331,R333,A354,S379,A382, see https://www.uniprot.org/uniprotkb/ P82251/variant-viewer), and active-site residues are colored in green. (d) Four other network scores (MSF, δλ, C5 and C12) for all residue positions based on the contact maps in (a).

The AlphaFold-predicted structure consists of a helical domain with deleterious mutations concentrating inside the core domain while those non-conserved residues are mostly located on the domain periphery (N-terminal and C-terminal helices) (see Fig 3C). The active site consists of residues 43–47 and 233 and is also located in the core domain (colored green in Fig 3C). The contact maps predicted by three coevolution analysis tools are all similar to the contact map based on the AlphaFold structure (see Fig 3A) (except that RaptorX omitted some local contacts in residues 1–200). As a result, the network scores agree well between these methods (see Fig 3B). Reassuringly, those residues identified with high network scores are primarily within the central core and overlap with most deleterious mutations (see a cluster of large red balls in Fig 3C), while the peripheral regions feature low scores consistent with low sequence conservation (see a cluster of small blue balls in Fig 3C). Among those top-10% predicted hotspot residues (see Fig 3C), mutations I44T, A126T, and W230R were implicated in Cystinuria. *In vitro* measurements showed W230R has almost no transport activity, and it was proposed that W230 serves as a gate between two substrate-binding pockets and undergoes conformational changes to enable amino acid transport [59]. Although the A126T mutation is mildly dysfunctional [60], it is notable among a cluster of conserved residues with small sidechains in the contact regions of transmembrane helices, hinting for its possible role in helix-helix association and relative motions.

#### Lipoprotein lipase

(Uniprot: P06858): As a key enzyme in triglyceride metabolism, it catalyzes the hydrolysis of triglycerides from circulating chylomicrons and very low density lipoproteins, thus playing an important role in lipid clearance from the blood stream, lipid utilization and storage (see https://www.uniprot.org/uniprotkb/P06858/entry#function).

**Fig 4:**
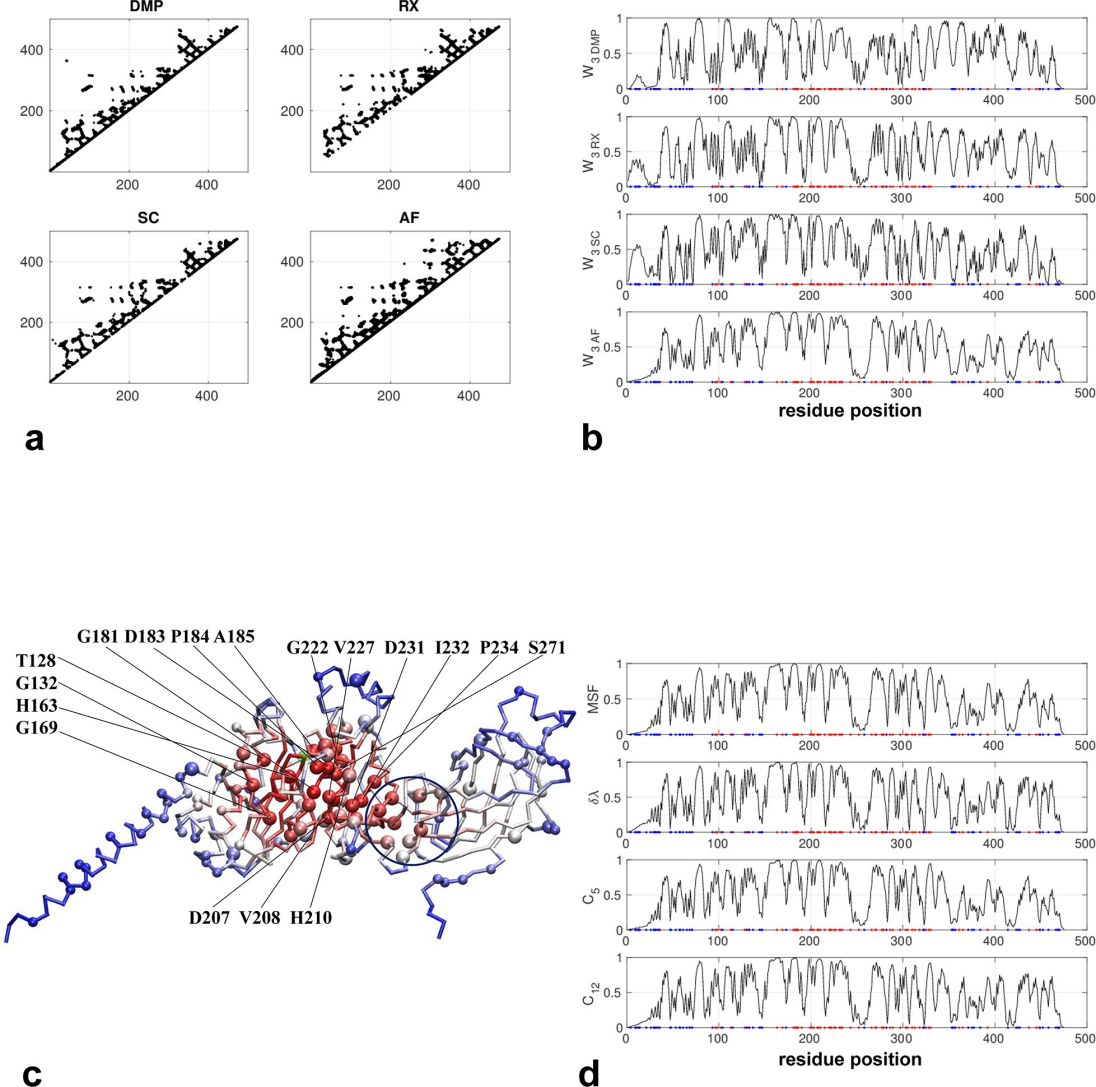
Results for lipoprotein lipase (Uniprot id: P06858). (a) Four contact maps constructed from coevolution analysis by DeepMetaPSICOV (DMP), RaptorX (RX), SPOT-Contact (SC), and the predicted structure by AlphaFold (AF) (only those contacts with probability >0.1 are shown). (b) W_3_ scores for all residue positions based on the contact maps in (a), where red and blue dots mark residues with deleterious and neutral mutations, respectively. (c) Predicted structure by AlphaFold as colored by W_3_ scores (red/blue for high/low values), where residues with deleterious and neutral mutations are shown as large and small balls, respectively (H71,A427,D36,N70,V96,A98,R102,W113,T128,G132,H163,G169,G181,D183,P184,A185, G186,E190,S199,D201,A203,D207,V208,H210,G215, S220,I221,G222, K225,V227,D231, I232,P234,C243,I252,C266,R270,S271,D277,S278,L279,S286,Y289,F297,L303,C305, C310,L313,N318,S325,M328,L330,A361,S365,L392,E437,E437,C445,E448, see https://www.uniprot.org/uniprotkb/P06858/variant-viewer), and active-site residues are colored in green. (d) Four other network scores (MSF, δλ, C5 and C12) for all residue positions based on the contact maps in (a).

The AlphaFold-predicted structure consists of an N-terminal helix, a central α/β domains, and a C-terminal β domain. Most deleterious mutations are concentrated inside the central domain while the non-conserved residues are mostly located on the periphery (including N-terminal helix and C-terminal domain) (see Fig 4C). The active site is comprised of a catalytic triad of S159, D183, and H268 [61] in the central domain (colored green in Fig 4C). The contact maps predicted by three coevolution analysis methods are similar to the contact map based on the AlphaFold structure (see Fig 4A). As a result, the network scores agree well between these methods (see Fig 4B) with minor differences in peripheral regions (such as the N-terminal helix). As predicted, those residues identified with high network scores are primarily within the central domain overlapping with most deleterious mutations (see a cluster of large red balls in Fig 4C), while the peripheral N-terminal helix and C-terminal domain feature low scores consistent with low sequence conservation (see a cluster of small blue balls in Fig 4C). Notably, some of them are found at the interface between the central domain and the C-terminal domain (circled in Fig 4C), possibly mediating inter-domain motions. Among those top-10% predicted hotspot residues (see Fig 4C), T128, G132, H163, G169, G181, D183, P184, A185, D207, V208, H210, G222, V227, D231, I232, P234 and S271 are known to harbor pathogenic mutations in Hyperlipoproteinemia 1, an autosomal recessive metabolic disorder characterized by defective breakdown of dietary fats. Both H163 and G169 lie in helix 4 that constitutes part of the highly conserved beta-epsilon serine-alpha folding motif which is near S159 of the active site. Supporting their functional relevance, mutations H163R and G169E were found to abolish the enzymatic activity [62]. Near D183 (one of the catalytic triad), mutations G181S and P184R were found to abolish the catalytic activity [63]. Further from D183, conserved substations D207E and H210Q abolished the enzyme activity [64], and mutations D231E, I232S and P234L led to loss of the catalytic function [65]. These mutations may disrupt allosteric interactions with the central catalytic domain. Another conservative mutation S271T (near D183) also led to loss of enzyme activity [66]. Taken together, these residues may function by directly or indirectly coupling to the active site.

## Conclusion

To conclude, we have integrated state-of-the-art machine learning, sequence coevolution analysis, network centrality/dynamics analysis, and deep-learning-powered protein structure prediction and protein language models to develop a protein variant site prediction method based on the protein residue contact networks which incorporate sequential, structural, dynamic, and interaction information simultaneously. Here is a summary of our contributions:

1. We build protein residue networks by exploiting three state-of-the-art coevolution analysis tools (RaptorX, DeepMetaPSICOV, and SPOT-Contact) that complement each other using different deep learning architectures. They make high-quality predictions of residue contact networks on par with the structures-based predictions by AlphaFold.
2. We use three popular machine learning algorithms (Random Forest, Gradient Boosting, and Extreme Gradient Boosting) to optimally combine 20 network centrality and dynamic scores to accurately predict key residue positions as hot spots for disease mutations. Despite their diversity in performance, the top-performing scores are highly correlated and have captured a common aspect of node importance (i.e. high degree of local connectivity).
3. We train and validate our method using a diverse dataset of 107 proteins rich in disease mutations, demonstrating its high accuracy in distinguishing between deleterious and neutral sites (with AUC of ROC ~ 0.84). The prediction quality is comparable with the state-of-the-art performance by ESM. Further marked improvement has been achieved after combining our method with the ESM method.

This study has established a useful strategy (i.e. ensemble learning) of combining an ensemble of diverse scores based on different coevolution analysis methods via machine learning to predict key variant sites of relevance to disease mutations. The code and dataset are made available to public to enable future developments and applications (see https://simtk.org/projects/hotspots).

For future work, it will be interesting to go beyond contact map predictions by integrating more sophisticated scores derived from the co-evolution analysis (for example, see refs [67–69]) in our workflow, which may further boost the accuracy of variant site prediction.

## Supporting information

S1 Table Evaluation of 20 network scores based on protein residue contact maps constructed from 3 coevolution analysis tools (DeepMetaPSICOV, RaptorX, and SPOT-Contact). (DOCX)

S2 Table Pearson correlations between network scores (row 1, 2, 3 and 4 correspond to results of DeepMetaPSICOV, SPOT-Contact, RaptorX and AlphaFold, respectively). (DOCX)
