# Supplementary material for: Predicting hotspots for disease-causing single nucleotide variants using sequences-based coevolution, network analysis, and machine learning
Source: PLoS One. 2024 May 14;19(5):e0302504. doi: 10.1371/journal.pone.0302504 (PMC11093321; doi:10.1371/journal.pone.0302504)
Supplement: S1 Table — (DOCX) [file pone.0302504.s001.docx]

**S1 Table.** **Evaluation of 20 network scores based on protein residue contact maps constructed from 3 coevolution analysis tools (DeepMetaPSICOV, RaptorX, and SPOT-Contact)**

| Score | AUC* of  DeepMetaPSICOV | AUC* of  RaptorX | AUC* of  SPOT-Contact |
| --- | --- | --- | --- |
| C1  C2  C3  C4  C5  C6  C7  C8  C9  C10  C11  C12  C13  δλ  MSF  W_1_  W_2_  W_3_  W_∞_  W_s_ | 0.75±0.13  0.75±0.17  0.78±0.10  0.65±0.12  0.78±0.17  0.63±0.16  0.77±0.11  0.65±0.12  0.79±0.13  0.76±0.12  0.80±0.13  0.78±0.16  0.75±0.17  0.80±0.13  0.81±0.14  0.81±0.14  0.81±0.15  0.81±0.14  0.80±0.13  0.80±0.13 | 0.78±0.14  0.75±0.19  0.75±0.15  0.52±0.15  0.78±0.17  0.56±0.19  0.61±0.20  0.52±0.15  0.78±0.16  0.75±0.14  0.78±0.17  0.80±0.14  0.74±0.18  0.78±0.14  0.79±0.15  0.79±0.15  0.77±0.15  0.78±0.15  0.77±0.14  0.78±0.13 | 0.75±0.15  0.76±0.19  0.70±0.15  0.61±0.07  0.78±0.18  0.65±0.17  0.72±0.16  0.61±0.07  0.75±0.16  0.69±0.15  0.78±0.17  0.80±0.17  0.76±0.19  0.78±0.16  0.80±0.17  0.80±0.17  0.79±0.17  0.80±0.16  0.78±0.16  0.78±0.16 |

* mean ± standard-deviation
