# Supplementary material for: Predicting hotspots for disease-causing single nucleotide variants using sequences-based coevolution, network analysis, and machine learning
Source: PLoS One. 2024 May 14;19(5):e0302504. doi: 10.1371/journal.pone.0302504 (PMC11093321; doi:10.1371/journal.pone.0302504)
Supplement: S2 Table — (DOCX) [file pone.0302504.s002.docx]

**S2 Table. Pearson correlations between network scores (row 1, 2, 3 and 4 correspond to results of DeepMetaPSICOV, SPOT-Contact, RaptorX and AlphaFold, respectively)**

|  | C1 C2 C3 C4 C5 C6 C7 C8 C9 C10 C11 C12 C13 W_1_ W_2_ W_3_ W_∞_ W_s_ MSF δλ |
| --- | --- |
| C1 C2 C3 C4 C5 C6 C7 C8 C9 C10 C11 C12 C13 W_1_ W_2_ W_3_ W_∞_ W_s_ MSF | 0.57 0.74 0.63 0.72 0.40 0.82 0.63 0.84 0.79 0.88 0.84 0.54 0.87 0.85 0.83 0.80 0.85 0.87 0.87  0.57 0.43 0.21 0.75 0.18 0.47 0.21 0.66 0.42 0.67 0.76 0.98 0.65 0.72 0.75 0.80 0.71 0.65 0.65  0.74 0.43 0.64 0.63 0.36 0.72 0.64 0.73 0.96 0.81 0.57 0.43 0.63 0.63 0.63 0.61 0.64 0.64 0.63  0.63 0.21 0.64 0.34 0.42 0.64 1.00 0.40 0.65 0.53 0.37 0.21 0.46 0.42 0.40 0.38 0.43 0.47 0.46  0.72 0.75 0.63 0.34 0.46 0.67 0.34 0.74 0.58 **0.91 0.86** 0.76 **0.80 0.83 0.84 0.84 0.83 0.80 0.80**  0.40 0.18 0.36 0.42 0.46 0.63 0.42 0.21 0.29 0.45 0.33 0.20 0.45 0.41 0.38 0.34 0.42 0.46 0.45  0.82 0.47 0.72 0.64 0.67 0.63 0.64 0.73 0.71 0.80 0.68 0.46 0.76 0.75 0.74 0.71 0.76 0.77 0.76  0.63 0.21 0.64 1.00 0.34 0.42 0.64 0.40 0.65 0.53 0.37 0.21 0.46 0.42 0.40 0.37 0.43 0.47 0.46  0.84 0.66 0.73 0.40 0.74 0.21 0.73 0.40 0.79 0.85 0.79 0.63 0.77 0.81 0.82 0.82 0.81 0.78 0.77  0.79 0.42 0.96 0.65 0.58 0.29 0.71 0.65 0.79 0.79 0.59 0.41 0.65 0.65 0.64 0.63 0.66 0.66 0.65  0.88 0.67 0.81 0.53 0.91 0.45 0.80 0.53 0.85 0.79  **0.85** 0.68 **0.84 0.85 0.85 0.84 0.86 0.85 0.84**  0.84 0.76 0.57 0.37 0.86 0.33 0.68 0.37 0.79 0.59 0.85 0.74 **0.91 0.94 0.94 0.92 0.92 0.90 0.91**  0.54 0.98 0.43 0.21 0.76 0.20 0.46 0.21 0.63 0.41 0.68 0.74 0.63 0.69 0.73 0.78 0.69 0.63 0.63  0.87 0.65 0.63 0.46 0.80 0.45 0.76 0.46 0.77 0.65 0.84 0.91 0.63  **0.98 0.97 0.93 0.98 1.00 1.00**  0.85 0.72 0.63 0.42 0.83 0.41 0.75 0.42 0.81 0.65 0.85 0.94 0.69 0.98  **1.00 0.97 1.00 0.99 0.98**  0.83 0.75 0.63 0.40 0.84 0.38 0.74 0.40 0.82 0.64 0.85 0.94 0.73 0.97 1.00  **0.99 0.99 0.97 0.97**  0.80 0.80 0.61 0.38 0.84 0.34 0.71 0.37 0.82 0.63 0.84 0.92 0.78 0.93 0.97 0.99  **0.97 0.93 0.93**  0.85 0.71 0.64 0.43 0.83 0.42 0.76 0.43 0.81 0.66 0.86 0.92 0.69 0.98 1.00 0.99 0.97  **0.99 0.98**  0.87 0.65 0.64 0.47 0.80 0.46 0.77 0.47 0.78 0.66 0.85 0.90 0.63 1.00 0.99 0.97 0.93 0.99  **1.00** |
| C1 C2 C3 C4 C5 C6 C7 C8 C9 C10 C11 C12 C13 W_1_ W_2_ W_3_ W_∞_ W_s_ MSF | 0.58 0.68 0.55 0.73 0.53 0.85 0.55 0.86 0.73 0.90 0.74 0.57 0.80 0.78 0.77 0.67 0.80 0.82 0.80  0.58 0.44 0.20 0.88 0.40 0.51 0.20 0.61 0.38 0.74 0.82 0.99 0.70 0.76 0.79 0.88 0.74 0.68 0.70  0.68 0.44 0.61 0.55 0.38 0.69 0.61 0.73 0.96 0.78 0.42 0.45 0.50 0.52 0.53 0.52 0.55 0.56 0.50  0.55 0.20 0.61 0.29 0.46 0.63 1.00 0.37 0.62 0.49 0.26 0.20 0.37 0.35 0.34 0.30 0.37 0.40 0.37  0.73 0.88 0.55 0.29 0.57 0.67 0.29 0.71 0.49 **0.88 0.91** **0.87 0.84 0.88 0.89 0.87 0.87 0.82 0.84**  0.53 0.40 0.38 0.46 0.57 0.73 0.46 0.33 0.31 0.54 0.55 0.40 0.64 0.61 0.59 0.49 0.62 0.66 0.64  0.85 0.51 0.69 0.63 0.67 0.73 0.63 0.75 0.68 0.82 0.66 0.51 0.76 0.75 0.73 0.64 0.77 0.80 0.76  0.55 0.20 0.61 1.00 0.29 0.46 0.63 0.37 0.62 0.49 0.26 0.20 0.37 0.35 0.34 0.30 0.37 0.40 0.37  0.86 0.61 0.73 0.37 0.71 0.33 0.75 0.37 0.77 0.87 0.66 0.59 0.68 0.71 0.72 0.68 0.73 0.71 0.68  0.73 0.38 0.96 0.62 0.49 0.31 0.68 0.62 0.77 0.76 0.38 0.38 0.47 0.49 0.49 0.47 0.52 0.54 0.47  0.90 0.74 0.78 0.49 0.88 0.54 0.82 0.49 0.87 0.76 **0.79** 0.73 **0.80 0.82 0.83 0.78 0.83 0.82 0.80**  0.74 0.82 0.42 0.26 0.91 0.55 0.66 0.26 0.66 0.38 0.79 0.80 **0.91 0.93 0.92 0.83 0.90 0.86 0.91**  0.57 0.99 0.45 0.20 0.87 0.40 0.51 0.20 0.59 0.38 0.73 0.80 0.69 0.75 0.78 0.88 0.73 0.67 0.69  0.80 0.70 0.50 0.37 0.84 0.64 0.76 0.37 0.68 0.47 0.80 0.91 0.69  **0.98 0.97 0.84 0.98 0.98 1.00**  0.78 0.76 0.52 0.35 0.88 0.61 0.75 0.35 0.71 0.49 0.82 0.93 0.75 0.98  **1.00 0.90 1.00 0.97 0.98**  0.77 0.79 0.53 0.34 0.89 0.59 0.73 0.34 0.72 0.49 0.83 0.92 0.78 0.97 1.00  **0.92 0.99 0.96 0.97**  0.67 0.88 0.52 0.30 0.87 0.49 0.64 0.30 0.68 0.47 0.78 0.83 0.88 0.84 0.90 0.92  **0.90 0.86 0.84**  0.80 0.74 0.55 0.37 0.87 0.62 0.77 0.37 0.73 0.52 0.83 0.90 0.73 0.98 1.00 0.99 0.90  **0.98 0.98**  0.82 0.68 0.56 0.40 0.82 0.66 0.80 0.40 0.71 0.54 0.82 0.86 0.67 0.98 0.97 0.96 0.86 0.98  **0.98** |
| C1 C2 C3 C4 C5 C6 C7 C8 C9 C10 C11 C12 C13 W_1_ W_2_ W_3_ W_∞_ W_s_ MSF | 0.63 0.58 0.22 0.64 0.22 0.29 0.22 0.75 0.65 0.71 0.78 0.55 0.88 0.87 0.85 0.58 0.87 0.68 0.73  0.63 0.64 0.15 0.82 0.23 0.24 0.15 0.66 0.66 0.78 0.70 0.93 0.60 0.65 0.67 0.82 0.64 0.70 0.55  0.58 0.64 0.44 0.79 0.35 0.39 0.44 0.64 0.93 0.88 0.51 0.68 0.48 0.50 0.50 0.64 0.50 0.69 0.47  0.22 0.15 0.44 0.32 0.70 0.67 1.00 0.11 0.38 0.39 0.06 0.22 0.18 0.14 0.14 0.30 0.15 0.37 0.17  0.64 0.82 0.79 0.32 0.45 0.43 0.32 0.65 0.72 **0.96** 0.71 **0.86** 0.63 0.65 0.66 0.79 0.65 **0.81** 0.62  0.22 0.23 0.35 0.70 0.45 0.70 0.70 0.12 0.25 0.45 0.17 0.29 0.32 0.28 0.26 0.40 0.28 0.51 0.31  0.29 0.24 0.39 0.67 0.43 0.70 0.67 0.34 0.34 0.48 0.28 0.29 0.30 0.28 0.27 0.40 0.29 0.49 0.39  0.22 0.15 0.44 1.00 0.32 0.70 0.67 0.11 0.38 0.39 0.06 0.22 0.18 0.14 0.14 0.30 0.15 0.37 0.17  0.75 0.66 0.64 0.11 0.65 0.12 0.34 0.11 0.71 0.72 0.71 0.61 0.66 0.69 0.69 0.62 0.69 0.66 0.64  0.65 0.66 0.93 0.38 0.72 0.25 0.34 0.38 0.71 0.83 0.55 0.67 0.52 0.53 0.53 0.66 0.54 0.72 0.51  0.71 0.78 0.88 0.39 0.96 0.45 0.48 0.39 0.72 0.83 0.68 **0.82** 0.64 0.65 0.65 0.77 0.65 **0.83** 0.63  0.78 0.70 0.51 0.06 0.71 0.17 0.28 0.06 0.71 0.55 0.68 0.64 0.76 0.77 0.77 0.63 0.77 0.67 0.70  0.55 0.93 0.68 0.22 0.86 0.29 0.29 0.22 0.61 0.67 0.82 0.64 0.53 0.58 0.61 0.85 0.58 0.72 0.52  0.88 0.60 0.48 0.18 0.63 0.32 0.30 0.18 0.66 0.52 0.64 0.76 0.53  **0.98 0.97** 0.69 **0.98** **0.81 0.85**  0.87 0.65 0.50 0.14 0.65 0.28 0.28 0.14 0.69 0.53 0.65 0.77 0.58 0.98  **0.99** 0.73 **1.00** **0.80 0.83**  0.85 0.67 0.50 0.14 0.66 0.26 0.27 0.14 0.69 0.53 0.65 0.77 0.61 0.97 0.99 0.75 **0.99** **0.80 0.82**  0.58 0.82 0.64 0.30 0.79 0.40 0.40 0.30 0.62 0.66 0.77 0.63 0.85 0.69 0.73 0.75 0.73 **0.88** 0.67  0.87 0.64 0.50 0.15 0.65 0.28 0.29 0.15 0.69 0.54 0.65 0.77 0.58 0.98 1.00 0.99 0.73 **0.81 0.83**  0.68 0.70 0.69 0.37 0.81 0.51 0.49 0.37 0.66 0.72 0.83 0.67 0.72 0.81 0.80 0.80 0.88 0.81 0.79 |
| C1 C2 C3 C4 C5 C6 C7 C8 C9 C10 C11 C12 C13 W_1_ W_2_ W_3_ W_∞_ W_s_ MSF | 0.87 0.77 0.38 0.95 0.42 0.82 0.38 0.90 0.81 0.96 0.98 0.85 0.97 0.97 0.96 0.86 0.97 0.95 0.97  0.87 0.78 0.26 0.91 0.32 0.81 0.26 0.98 0.79 0.90 0.89 0.99 0.87 0.91 0.93 0.99 0.91 0.91 0.87  0.77 0.78 0.52 0.79 0.37 0.74 0.52 0.82 0.97 0.83 0.72 0.78 0.71 0.74 0.75 0.78 0.74 0.79 0.71  0.38 0.26 0.52 0.33 0.61 0.55 1.00 0.30 0.51 0.39 0.30 0.27 0.30 0.30 0.29 0.27 0.30 0.33 0.30  0.95 0.91 0.79 0.33 0.46 0.85 0.33 0.92 0.79 **0.99 0.96** 0.91 **0.95 0.96 0.96 0.91 0.96 1.00 0.95**  0.42 0.32 0.37 0.61 0.46 0.67 0.61 0.33 0.31 0.46 0.40 0.33 0.41 0.39 0.37 0.33 0.39 0.46 0.41  0.82 0.81 0.74 0.55 0.85 0.67 0.55 0.83 0.72 0.85 0.81 0.81 0.80 0.82 0.83 0.81 0.82 0.84 0.80  0.38 0.26 0.52 1.00 0.33 0.61 0.55 0.30 0.51 0.39 0.30 0.27 0.30 0.30 0.29 0.27 0.30 0.33 0.30  0.90 0.98 0.82 0.30 0.92 0.33 0.83 0.30 0.84 0.93 0.91 0.96 0.89 0.93 0.95 0.97 0.93 0.92 0.89  0.81 0.79 0.97 0.51 0.79 0.31 0.72 0.51 0.84 0.84 0.75 0.79 0.73 0.77 0.78 0.79 0.77 0.80 0.73  0.96 0.90 0.83 0.39 0.99 0.46 0.85 0.39 0.93 0.84 **0.95** 0.90 **0.94 0.95 0.95 0.90 0.95 0.99 0.94**  0.98 0.89 0.72 0.30 0.96 0.40 0.81 0.30 0.91 0.75 0.95 0.88 **1.00 1.00 0.99 0.89 0.99 0.96 1.00**  0.85 0.99 0.78 0.27 0.91 0.33 0.81 0.27 0.96 0.79 0.90 0.88 0.85 0.90 0.92 0.99 0.90 0.91 0.85  0.97 0.87 0.71 0.30 0.95 0.41 0.80 0.30 0.89 0.73 0.94 1.00 0.85  **0.99 0.97 0.86 0.98 0.95 1.00**  0.97 0.91 0.74 0.30 0.96 0.39 0.82 0.30 0.93 0.77 0.95 1.00 0.90 0.99  **1.00 0.91 1.00 0.96 0.99**  0.96 0.93 0.75 0.29 0.96 0.37 0.83 0.29 0.95 0.78 0.95 0.99 0.92 0.97 1.00  **0.93 1.00 0.96 0.97**  0.86 0.99 0.78 0.27 0.91 0.33 0.81 0.27 0.97 0.79 0.90 0.89 0.99 0.86 0.91 0.93  **0.91 0.91 0.86**  0.97 0.91 0.74 0.30 0.96 0.39 0.82 0.30 0.93 0.77 0.95 0.99 0.90 0.98 1.00 1.00 0.91  **0.96 0.98**  0.95 0.91 0.79 0.33 1.00 0.46 0.84 0.33 0.92 0.80 0.99 0.96 0.91 0.95 0.96 0.96 0.91 0.96  **0.95** |
